# G-Proteins Agonists and NO/cGMP Blockers: Unexplored Frontiers in the Pharmaceutical Industry

**DOI:** 10.5935/abc.20170139

**Published:** 2017-10

**Authors:** Paulo Roberto B. Evora

**Affiliations:** Departamento de Cirurgia e Anatomia - Faculdade de Medicina de Ribeirão Preto - Universidade de São Paulo, São Paulo, SP - Brazil

**Keywords:** GTP-Binding Proteins, Nitric Oxide, Endothelial Cells, Technology Pharmaceutical, Reference Drugs

Two unexplored therapeutic frontiers the pharmaceutical industry still must address: 1) to answer the questions about the G-proteins’ signal transduction to keep the NO release normal, and 2) to block the amine-resistant inflammatory vasoplegia mediated by NO overproduction. It is important to remember that two Nobel prizes are involved.

## G-proteins: potential therapeutic role

The release of nitric oxide (NO) can occur by different pathways involving G-proteins. The Gi-protein is responsible for the mediation of inhibitory effects of receptors in the adenylate cyclase and guanylate cyclase pathways. An early stage of the majority of the responses mediated by receptors is the activation of G-proteins in the cell membrane, which is the target of the modulation of a variety of intracellular events. The role of G-proteins in the pathophysiology of vasospasm after global ischemia and reperfusion is still a matter of investigations. Their participation was documented in a comparative study of vascular relaxation induced by sodium fluoride, which produces biphasic responses in human, bovine, and porcine coronary arteries, causing an endothelium-dependent relaxation and an endothelium-independent contraction. G-protein dysfunction in the endothelium has also been postulated as responsible for the endothelial dysfunction in conditions of endothelial cell regeneration after injury, atherosclerosis, and coronary vasospasm. Myocardial ischemia and reperfusion selectively impair receptor-mediated NO release. However, the ability of the endothelial cell to produce NO or generate endothelium-dependent relaxation to nonnitric oxide-dependent agonists remains intact.^1,2^

In summary: 1. Endothelial cells maintain their capacity to release NO based on their ability in receiving the transduction signal through the membrane; 2. G-proteins have a fundamental role in the signal transduction; 3. This paradigm is extended to all vasotonic cardiovascular diseases that coexist with platelet dysfunction. These data would be highly relevant in the research of G-protein-targeting drugs.

## The cGMP/cAMP “crosstalk” is underestimated

At present, clinical management of inflammatory vasoplegia associated with sepsis or anaphylaxis is symptomatic. Volume is expanded using administration of fluids, and low blood pressure is managed using administration of positive inotropes and vasoconstrictors. However, circulatory shock is frequently refractory to high amine concentrations.

Since 1994, blockade of guanylate cyclase by methylene blue (MB) in distributive shock has been the subject of study in our Laboratory of Endothelial Function and has been clinically used by the Cardiovascular Surgery Group, both at Ribeirao Preto Medical School of the University of Sao Paulo (FMRP-USP). There is strong evidence that MB, a guanylate cyclase inhibitor, is a therapeutic option for the treatment of the vasoplegic syndrome. Based on our clinical and laboratory experience, accumulated over a period of 20 years, classic concepts about the use of MB in this condition have been established: 1) Heparin and ACE inhibitors are risk factors; 2) At recommended doses, MB is considered a safe drug (the lethal dose is 40 mg/kg); 3) MB does not cause endothelial dysfunction; 4) The effects of MB appear only in the case of nitric oxide (NO) upregulation; 5) MB is not a vasoconstrictor *per se*; by blocking the cGMP system, it "releases" the cAMP system in a kind of “crosstalk”, facilitating the vasoconstrictor effect of noradrenaline; 6) The most commonly used dosage is 2 mg/kg intravenous bolus followed by continuous infusion, since plasma concentration decreases markedly in the first 40 minutes; 7) There is a possible "window of opportunity" for the effectiveness of the MB.^3-5^

In this milieu, one main question comes up: ‘What can we do when circulatory shock becomes refractory to the classical therapeutic measures including fluid administration, inotropes, and vasoconstrictors? Responses to this question are currently limited to the accumulated evidence regarding three cAMP-independent vasoconstriction mechanisms: 1) cGMP/NO-dependent vasoconstriction (the most important mechanism); 2) vasopressin administration and; 3) hyperpolarization-dependent vasoconstriction. Why these therapeutic alternatives do not always work?’ We believe that there are at least, five aspects pertaining to this inquiry: 1) The lack of consideration of existing ‘guidelines’ or ‘evidence based medicine’ regarding the accepted treatment options available; 2) lack of knowledge of different vasodilatation mechanisms; 3) the possibility of a crosstalk between different vasodilatation mechanisms; 4) the soluble guanylyl cyclase (sGC) enzymatic activity and; 5) the common use of MB as a ‘rescue’ or ‘ultimate’ therapeutic attempt.^6^

Although there are no definitive multicentric studies, the use of MB is currently the unique, safest, cheapest treatment option for vasoplegic syndrome in cardiac surgery. Nevertheless, the MB "affair” masks the real problem of vasoplegic endothelial dysfunction, whose blockade could be the target of current drugs other than MB.

However, in the scope of an editorial, it must be considered that there is no simple answer to the questions addressed above, since there are multiple factors that influence the decision making in multimillion dollar investments. Even considering the actual and potentialy clinical benefits, one must consider the patent situation of the product and its development, as well as the potential of present and future market. In addition, according to executives of the pharmaceutical industry, there is also a possible competition for funding that often entails internal competition between many lines of research.

**Figure 1:**
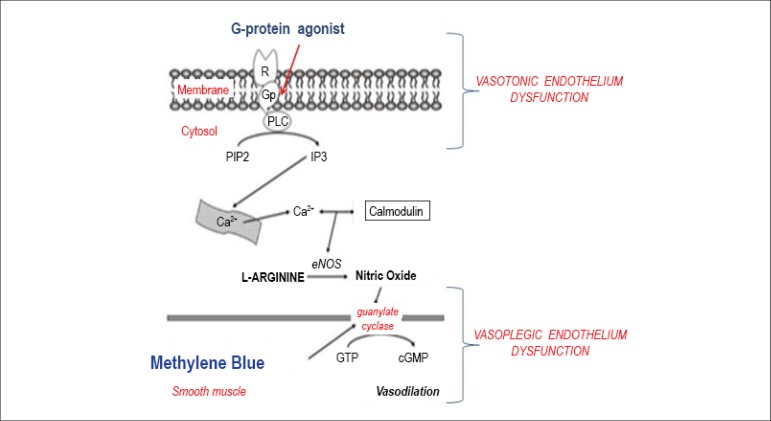
Endothelial nitric oxide synthase converts L-arginine to nitric oxide, which activates guanylate cyclase, responsible for the conversion of GTP to cGMP that causes endothelium-dependent vasodilatation commonly associated with circulatory shock mediated by membrane receptors (Adapted from Evora & Simon; Ann Allergy Asthma Immunol. 2007;99:306-313.)^7^

These considerations would be speculative, but in our opinion the pharmaceutical industry owes us explanations on: 1) questions about the G-proteins signal transduction to keep NO release normal, and 2) blockage of the amine-resistant inflammatory vasoplegia mediated by NO overproduction. It is important to remember that two Nobel prizes are involved (Figure 1).
